# Quinazolinone-Amino Acid Hybrids as Dual Inhibitors of EGFR Kinase and Tubulin Polymerization

**DOI:** 10.3390/molecules23071699

**Published:** 2018-07-12

**Authors:** Mohamed F. Zayed, Heba S. Rateb, Sahar Ahmed, Osama A. Khaled, Sabrin R. M. Ibrahim

**Affiliations:** 1 Department of Pharmacognosy and Pharmaceutical Chemistry, College of Pharmacy, Taibah University, Al-Madinah 41477, Al-Munawarah, Saudi Arabia; smahmed@taibahu.edu.sa (S.A.); sribrahim@taibahu.edu.sa (S.R.M.I.); 2 Department of Pharmaceutical Chemistry, Faculty of Pharmacy, Al-Azhar University, Cairo 11884, Egypt; 3 Department of Pharmaceutical and Medicinal Chemistry, Pharmacy College, Misr University for Science and Technology, Cairo 12568, Egypt; 4 Department of Medicinal Chemistry, Faculty of Pharmacy, Assiut University, Assuit 71526, Egypt; 5 Department of Physical Therapy, College of Medical Rehabilitation Sciences, Taibah University, Al-Madinah 41477, Al-Munawarah, Saudi Arabia; drosama79@gmail.com; 6 Department of Basic Science, Faculty of Physical Therapy, Cairo University, Cairo 12613, Egypt; 7 Department of Pharmacognosy, Faculty of Pharmacy, Assiut University, Assiut 71526, Egypt

**Keywords:** synthesis, fluoroquinazolinones, antitumor, EGFR inhibitor, tubulin inhibitor

## Abstract

Some fluoroquinazolinones (**A**–**H**) were designed, synthesized and biologically evaluated for their antitumor activity against the two cell lines, MCF-7 and MDA-MBA-231. New derivative **G** (IC_50_ = 0.44 ± 0.01 µM) showed antitumor activity, better than that of the reference drug erlotinib (IC_50_ = 1.14 ± 0.04 µM) against MCF-7. New derivative **E** (IC_50_ = 0.43 ± 0.02 µM) showed higher activity than the reference drug erlotinib (IC_50_ = 2.55 ± 0.19 µM) against MDA-MBA-231. Furthermore, the EGFR (epidermal growth factor receptor) and tubulin inhibition assays were carried out for the highest active derivatives to reveal the expected mechanism of action. They exhibited significant results compared to the reference drugs. Molecular docking simulations were performed on EGFR and tubulin binding sites to rationalize the experimental results and describe their binding modes. The results of the molecular modeling study were correlated with that of the antitumor screening.

## 1. Introduction

**Figure 1:**
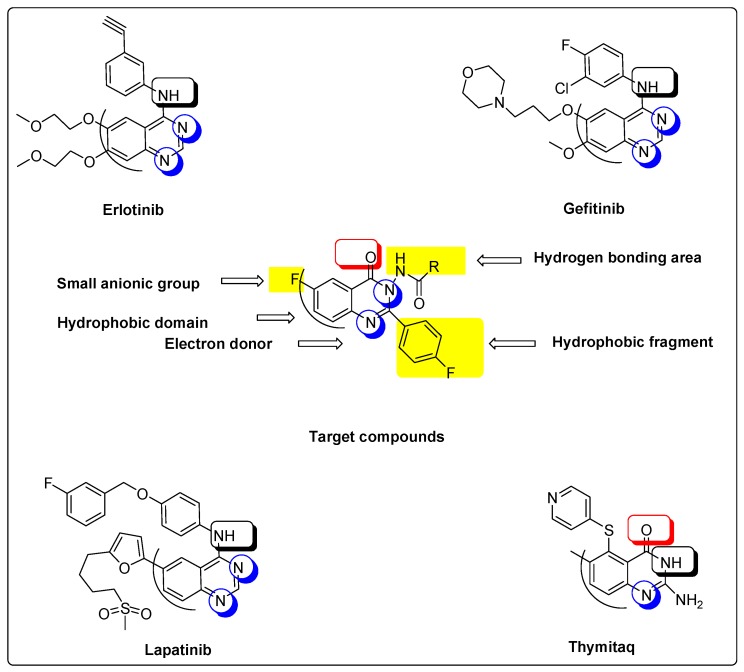
Structures of the newly-synthesized quinazolinones comparing to the reference antitumor drugs.

Quinazolines are a significant group of heterocyclic derivatives possessing a broad variety of biological activities [1,2,3,4,5,6,7,8,9,10,11]. The antitumor activity of quinazoline derivatives is well-known [12,13,14,15,16,17]. Many quinazoline derivatives, such as erlotinib, gefitinib and lapatinib, are used as antitumor agents, and target the epidermal growth factor receptor (EGFR) protein kinase [15,16]. Other antitumor quinazolines target thymidylate synthase, such as thymitaq [13]. Molecular study of these well-known drugs, displayed in Figure 1, found that all of these antitumor agents were formed of a quinazoline nucleus joined with different substituents at different positions. The quinazoline ring system is an aromatic heterocyclic system with a bicyclic structure, formed of a phenyl ring (hydrophobic domain) and a pyrimidine ring. The aromatic ring in the antitumor quinazolines is joined with different hydrophobic or hydrophilic substituents at positions 5, 6 and/or 7. The heterocyclic ring has different substituents at the 2 and/or 4 positions. We have reported in previous studies [13,16] that antitumor activity of some quinazoline derivatives was improved by halogenation of quinazoline at position 6 and hydrophobic substitution of the quinazoline nucleus with a phenyl ring at position 3. Moreover, it was reported that fluorine substitution might develop the whole pharmacokinetic and pharmacodynamic characters of the molecule by increasing selectivity, bioavailability, metabolic stability and binding interactions [18,19,20,21]. Taken together, our study is based on designing some new compounds with the previously mentioned features in addition to some new modifications that might enhance antitumor activity. The new compounds were designed as hybrid molecules, having the quinazolinone nucleus joined with different types of l-amino acids at position 3 to obtain a free amino group (NH_2_) or free carboxylic group (COOH) in the designed model. These groups could form extra hydrogen bonds with the receptor site. Therefore, better binding and better activity could be obtained. Amino acids were selected to include different hydrophobic and hydrophilic groups to study and compare their activity. All amino acids substitutions have a free primary amino group (NH_2_) except l-proline, which has a secondary amino group (NH) merged into a five-membered heterocyclic ring. These varied substitutions allow us to compare the effect of each amino group on the binding process. In addition, there are two fluoride atoms in the molecule to improve the overall pharmacokinetics and pharmacodynamics of this molecule. One of these two fluoride atoms is attached directly at position 6 of quinazolinone, while the other one is joined indirectly at position 2. Thus, this study presents a new model of quinazolinones as antitumor molecules, having a 4-flurophenyl group at position 2, different amino acids at position 3, and a fluoride group at position 6. This new model could form extra hydrogen bonds with the EGFR binding site to yield more active agents. Figure 1 displays molecular similarities between reference antitumor quinazolines and our target molecules. 

## 2. Results and Discussion

### 2.1. Chemistry

**Scheme 1:**
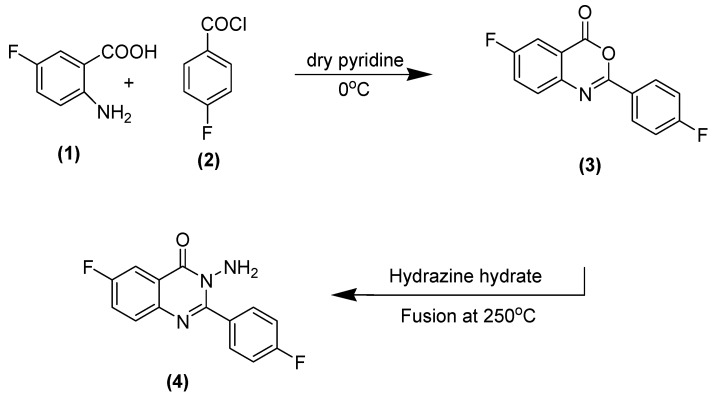
Synthesis of benzoxazinone (**3**) and quinazolinone (**4**).

**Scheme 2:**
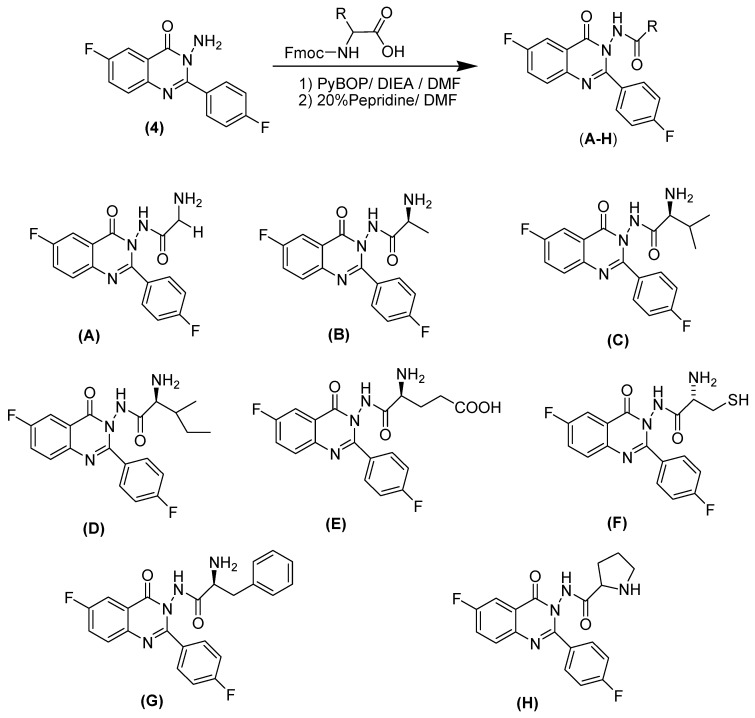
Synthesis of substituted fluoroquinazolinone derivatives (**A**–**H**).

The synthetic methodology of the title compounds of substituted quinazolinone (**A**–**H**) is explained in Scheme 1 and Scheme 2. It includes three types of reactions, including a benzoylation reaction of 2-amino-5-flurobenzoic acid (**1**) through reaction of a later compound with 4-flurobenzoylchloride (**2**) to produce a benzoxazinone derivative (**3**), and a nucleophilic displacement of the benzoxazinone derivative by fusion of this compound with hydrazine hydrate at 250 °C to get aminoquinazolinone (**4**). 

The third reaction is a coupling reaction of aminoquinazolinone (**4**) with different derivatives of Fmoc-protected l-amino acids, through the application of different electronic environments, followed by basic hydrolysis, to obtain the target compounds (**A**–**H**). The coupling step was performed in the presence of an effective coupling agent (PyBOP/DIEA) to help perform the reaction without racemization. Three kinds of amino acids were used, namely aliphatic, aromatic and heterocyclic amino acids. These different types of amino acids formed different molecules with different hydrophilic and hydrophobic groups, which could help strengthen ligand–receptor interactions by forming extra hydrogen bonds or strong hydrophobic interactions.

### 2.2. Cytotoxic Screening 

The antitumor screening was done using two cell lines: MCF-7 and MDA-MB-231. These two cell cultures are highly overexpressed in breast cancer patients, and they represent two different types: MCF-7 cells exhibit low invasiveness and are responsive to estrogen, whereas MDA-MB-231 cells are highly invasive and do not respond to estrogen [22]. The target derivatives (**A**–**H**) were exposed to cytotoxic screening against these two cell lines using an MTT assay [23,24]. The IC_50_ values are shown in Table 1.

**Figure 2:**
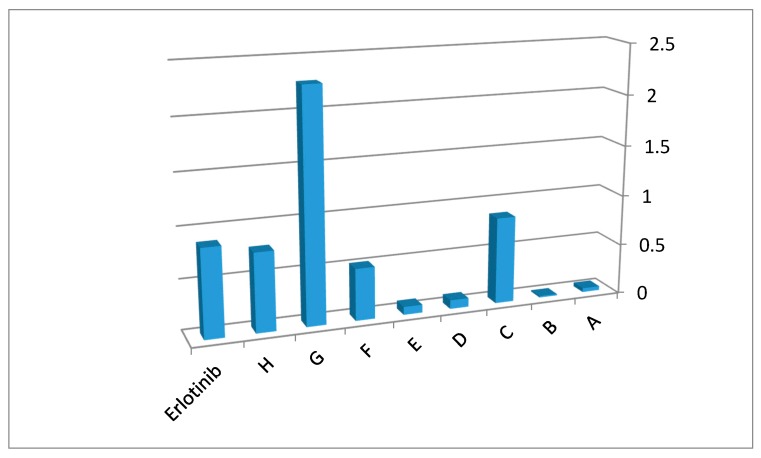
1/IC_50_ values of antitumor screening of target derivatives against MCF-7. It shows their activity in comparison to the reference erlotinib.

The IC_50_ values of the MCF-7 cell line show that all title compounds have good activity. Compound **G** displays better activity than the reference erlotinib against MCF-7 cell line. Compound **G** (IC_50_ = 0.44 ± 0.01 µM), the highest active compound, contains a l-phenylalanine substitution at position 3 of the quinazolinone nucleus. l-phenylalanine has a benzyl side chain. This side chain could form strong hydrophobic interactions with the binding site of erlotinib leading to increased binding affinity. Additionally, the high lipophilic characters of this entity, when compared to the other entities, might increase the antitumor activity. Compound **B** (IC_50_ = 68.49 ± 3.27 µM), the lowest active compound, contains l-alanine unit moiety with a methyl group. The low lipophilic characters of this unit, compared to other units, could lead to decreasing hydrophobic interactions and thus biological activity. The other compounds had mid-level activity, ranging from IC_50_ = 1.16 ± 0.05 µM to IC_50_ = 24.97 ± 1.61 µM. The activity of the target derivatives can be sorted as: **G** > **C** > **H** > **F** > **D** > **E** > **A** > **B**. Figure 2 displays the 1/IC_50_ values for the target compounds against the MCF-7 cell line, compared against the reference erlotinib.

**Figure 3:**
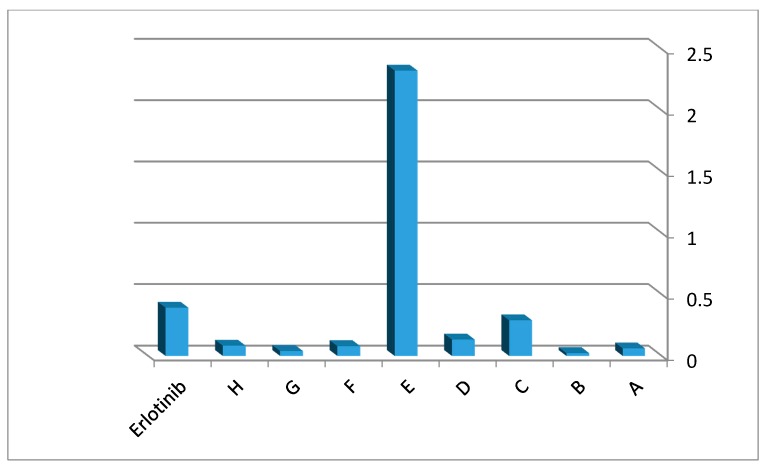
1/IC_50_ values of antitumor screening of target derivatives against the MDA-MBA-231 cell line. It shows their activity in comparison to the reference erlotinib.

**Figure 4:**
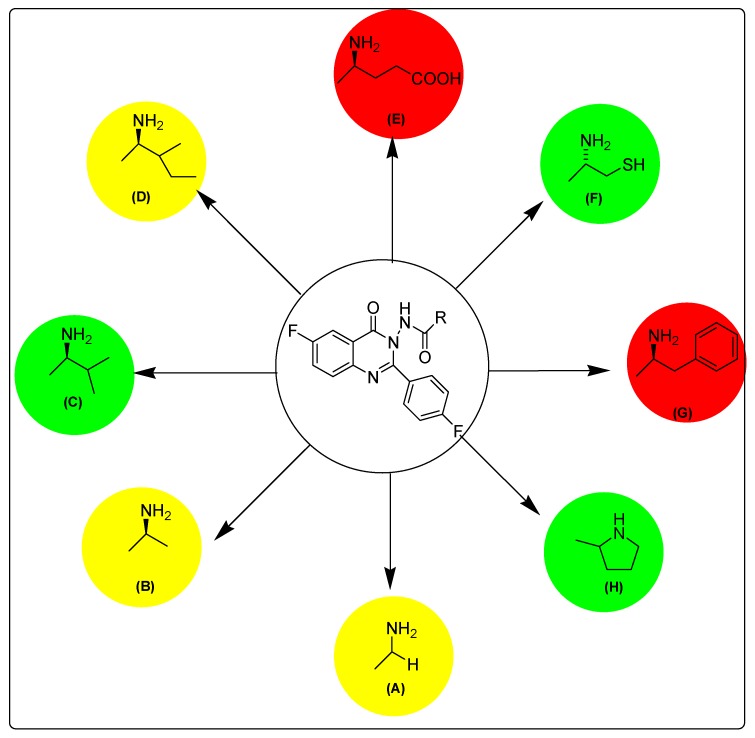
Structure activity relationship map of target compounds. Red indicates highly active derivatives, green indicates mild active derivatives and yellow indicates low active derivatives.

The IC_50_ values for the MDA-MBA-231 cell line shows good activity for all the derivatives, as depicted in Figure 3. Compound **E** had higher activity than erlotinib. The highest active compound, **E** (IC_50_ = 0.43 ± 0.02 µM), has l-glutamine at position 3 from quinazolinone. This substitution contains a free (NH_2_) group and two (COOH) groups that could form extra hydrogen bonds with the binding site of erlotinib. Hydrogen bonds lead to tight fitting and better binding of the ligand with the EGFR receptor site. Compound **B** (IC_50_ = 42.93 ± 2.64 µM) was the lowest active derivative for the MDA-MBA-231 cell line, and was also the lowest one against MCF-7. This could be explained based on the presence of a low lipophilic group (CH_3_) and low hydrophobic interaction between the ligand and receptor. The other compounds had mid-level activity, ranging from IC_50_ = 3.45 ± 0.21 µM to IC_50_ = 24.67 ± 1.7 µM. Cytotoxic action of the title compounds can be set in this manner: **E** > **C** > **D** > **H** > **F** > **A** > **G** > **B**. Figure 3 shows the 1/IC_50_ values against MDA-MBA-231 cell line compared to erlotinib, while Figure 4 shows the structure activity map of target compounds. Further molecular docking study was needed to justify the results and elucidate the way of binding of these derivatives with the receptor site.

**Figure 5:**
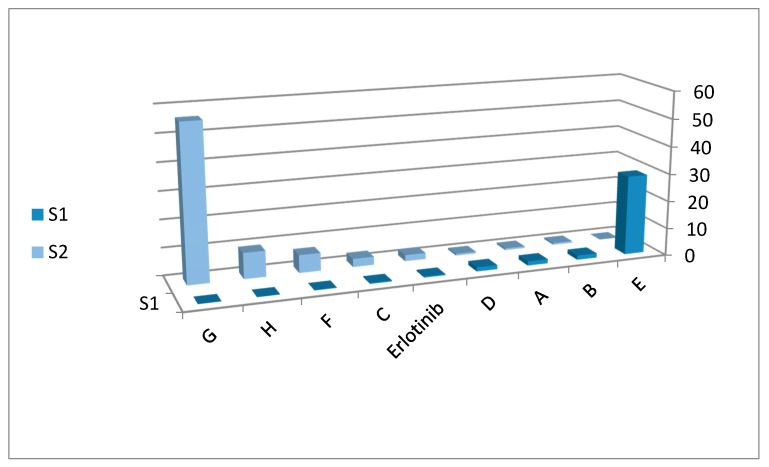
Selectivity of target derivatives against MCF-7 and MDA-MBA-231 cells. The greater the S1 value (dark blue), the greater the selectivity toward MDA-MBA-231; whereas the greater the S2 value (light blue), the greater the selectivity toward MCF-7.

The selectivity study determined that compounds with an ethylcarboxylate (**E**), methyl (**B**), hydrogen (**A**) or 2-methylbutane (**D**) substitution at position 3 were more selective to MDA-MBA-231 than MCF-7. On the other hand, compounds having a 2-methylpropane (**C**), ethanethiol (**F**), pyrrolidine (**H**) and benzyl (**G**) substitution at position 3 of substituted quinazolinone were more selective to MCF-7 than MDA-MBA-231. Table 2 and Figure 5 show selectivity of the title derivatives.

### 2.3. EGFR Assay

Epidermal growth factor receptors (EGFR) are overexpressed in most types of tumors. Consequently, these receptors are targeted by most antitumor drugs [15,16]. Several quinazolines like gefitinib, lapatinib, erlotinib and canertinib have great therapeutic potential in cancer treatment through inhibition of EGFR [23,24]. Based on the aforementioned facts and our previous study [16], EGFR inhibitory activity of the highest active compounds **G** (the highest active compound against MCF-7) and **E** (the highest active compound against MDA-MBA-231) was examined. Compound **G**, which contains a benzyl moiety, displayed strong epidermal growth factor receptor tyrosine kinase (EGFR-TK) inhibitory activity against MCF-7; while compound **E** showed strong inhibitory activity against MDA-MBA-231. Compound **G** had IC_50_ = 163.97 nM against MCF-7 cells. Compound **E** contains an ethylcarboxylate moiety, and had IC_50_ = 545.38 nM against MDA-MBA-231 cells. The reference erlotinib had IC_50_ = 78.04 nM against MCF-7 cells and 299 nM against MDA-MBA-231 cells, respectively. From the previous results, we notice the potency of these derivatives as EGFR inhibitors, but further investigations were necessary to explain their mode of binding with the EGFR binding site. Table 3 shows IC_50_ values for the epidermal growth factor receptor (EGFR) assay of compounds **G**, **E** and erlotinib. 

### 2.4. In Silico Screening and Molecular Docking Study into EGFR Binding Site 

In silico screening was performed based on the structure-based design (SBD) method using the Molecular Operating Environment (MOE) program, as described in the “Materials and Methods”. The docking experiments were performed using target derivatives on the experimental co-crystallized EGFR (Protein data bank PDB; 1M17), and the resulting lowest energies and scoring values are shown Table 4.

Based on the results displayed in Table 4, we notice that compound **E** has the following properties:

(1) It is the highest active compound among all the compounds.

(2) It has a unique structure, characterized by the presence of two hydrogen bonding groups: (NH_2_) and (COOH). They originate from l-glutamine, which was substituted into position 3 from quinazolinone, and thus were able to form the hydrogen bonds necessary to match our study goal. 

**Figure 6:**
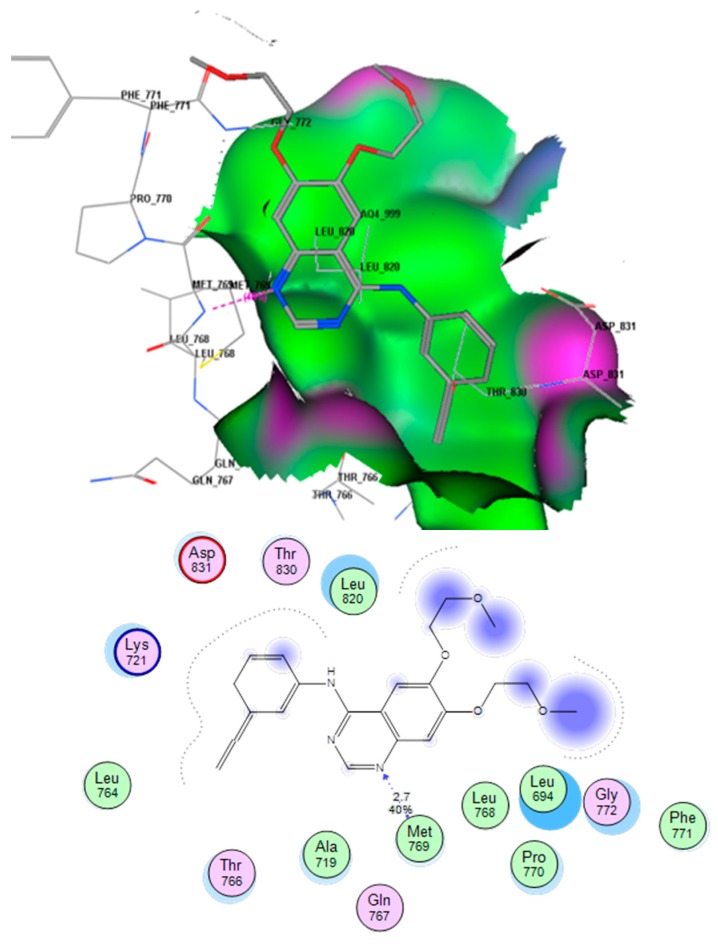
Interactions of erlotinib into EGFR binding site. Red color shows high polarity region, pink color shows mild polarity region and green color shows hydrophobic region with low polarity. The images show one type of hydrogen bonding.

This docking process was completed to identify the mode of binding of the title compounds with EGFR. Using the MOE software to compare the binding mode of compound **E** with that of erlotinib, it was found that compound **E** was docked inside the binding site of erlotinib in the crystalline structure of EGFR (PDB; 1M17). The co-crystallized erlotinib showed an H-bond between the N1 atom of quinazoline and the (OH) of Met769 (distance 3.19 Å). There were also some hydrophobic interactions for the two aromatic rings and the aliphatic side chain. Figure 6 shows these molecular interactions.

**Figure 7:**
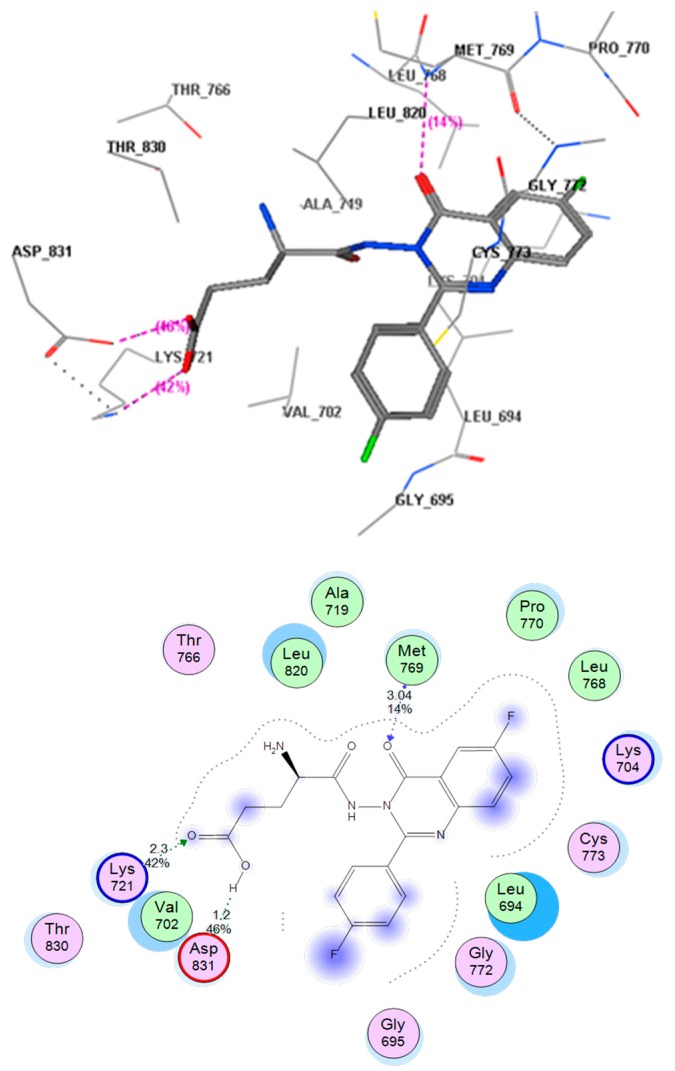
Interactions of compound **E** with the EGFR binding site. The red color shows a high polarity region, the pink color shows a mild polarity region and the green color shows a hydrophobic region with low polarity. The image shows three types of hydrogen bonds, as represented by dotted lines.

Title compound (**E**) was able to occupy the EGFR binding site in the same way, through formation of hydrogen bonds with Met769 (distance 3.04 Å) and the oxygen atom in (C=O) at position 4 of the quinazolinone moiety. Furthermore, there were two extra hydrogen bonds which made the ligand binding strength better than that of erlotinib. The first extra hydrogen bond was between the (OH) of the (COOH) group and the amino acid residue Asp831 (distance 1.2 Å). The second extra hydrogen bond was between the free oxygen atom of the (COOH) group and Lys721 (distance 2.3 Å). In addition, there were some hydrophobic interactions associated with the two aromatic rings and the two fluoride atoms. These interactions produced a better energy score (−23.5 Kcal/mol) for compound **E** than that of erlotinib (−13.5 Kcal/mol). Figure 7 shows the molecular interactions of compound **E** with the EGFR binding site.

### 2.5. Tubulin Polymerization Inhibition Assay

Inhibition of the tubulin polymerization process stops cellular mitotic divisions and, consequently, tubulin polymerization inhibition is one of the important targets in treatment of many types of cancer [25,26]. Many quinazoline derivatives have excellent activity as tubulin inhibitors [16,26]. These derivatives possess structural similarity with our target compounds. Based on this information, it was decided to detect the influence of these molecules on the tubulin polymerization process. The tubulin assay was detected kinetically using the CytoDYNAMIX Screen kit for the highest active compound, compound **E**, as described in the “Materials and Methods”. This assay showed IC_50_ = 6.24 µM for compound **E** and IC_50_ = 1.33 µM for the reference colchicine on the same cell line. This result reveals good activity for compound **E** as strong inhibitor of tubulin polymerization, and this may explain the high cytotoxic activity of these compounds. Further exploration for the binding interactions of these compounds with the tubulin binding site could be clarified by a molecular docking study.

### 2.6. In Silico Screening and Molecular Docking Study into Tubulin Binding Site

In silico screening was performed based on the structure-based design (SBD) method, using the MOE program as described in the “Materials and Methods”. The docking experiments were performed using target derivatives on the experimental co-crystallized tubulin (PDB; 1SA0), and the resulting lowest energies and scoring values are shown Table 5.

**Figure 8:**
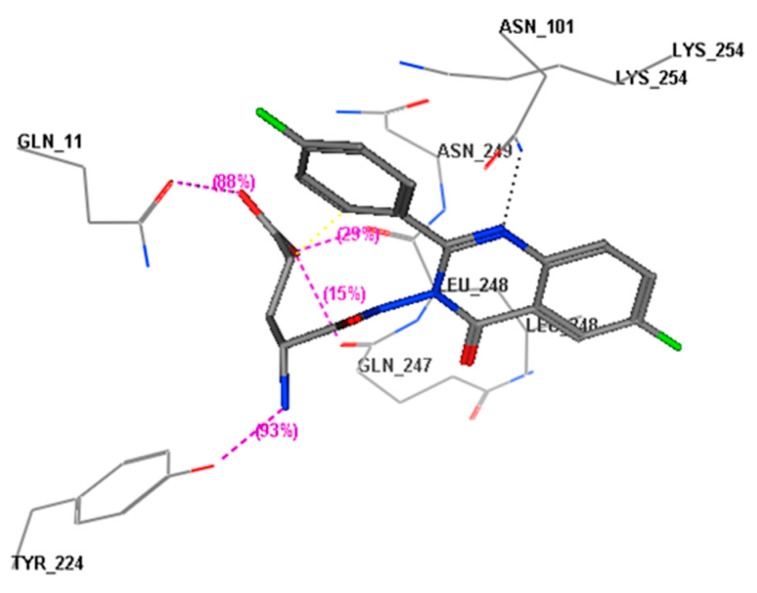

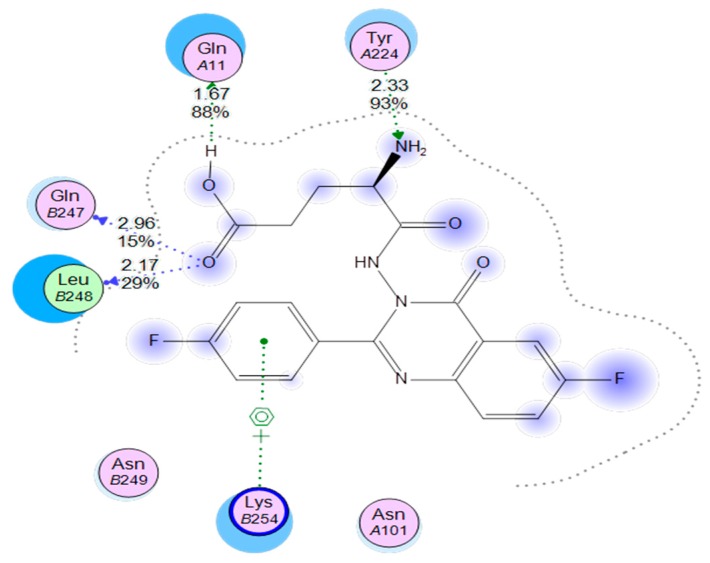
Compound **E** inside tubulin binding site. The images show four kinds of hydrogen bond interactions, represented by dotted lines.

Using the MOE software to compare the binding mode of compound **E** with that of colchicine, it was found that compound **E** was docked inside the binding site of colchicine in the crystalline structure of tubulin (PDB; 1SA0). When compound **E** docked into this binding site, it occupied that binding site in the same manner as colchicine, in addition to three extra hydrogen bonds. These bonds helped to strengthen the fit inside the receptor. Figure 8 demonstrates the binding interactions of compound **E** with the colchicine binding site, showing the formation of a strong hydrogen bond between the free NH_2_ amino group and the amino acid residue TyrA224 (distance 2.33 Å), a hydrogen bond between the (OH) of the COOH group and the amino acid residue GlnA111 (distance 1.67 Å), a hydrogen bond between the (C=O) of the COOH group and amino acid residue GlnB247 (distance 2.96 Å), and a hydrogen bond between the (C=O) of the COOH group and the amino acid residue LeuB248 (distance 2.17 Å). These interactions produced a better energy score (−24.7 Kcal/mol) for compound **E** than that of colchicine (−11.1 Kcal/mol).

## 3. Materials and Methods 

### 3.1. General

Chemicals were purchased from Sigma-Aldrich (St. Louis, MO, USA). Solvents were prepared according to standard methods. Aluminum sheets (Type 60 GF256) of pre-coated silica gel were used for TLC. Spots were detected by exposure to a UV-lamp at λ = 254 nm. All melting points were measured by Mel-Temp apparatus and they are uncorrected. IR spectra were measured by KBr discs and Perkin Elmer spectrophotometer (PerkinElmer, Melville, NY, USA). ^1^HNMR and ^13^CNMR spectra were detected by Bruker FT-NMR/400 (400 MHz) (Bruker BioSpin, Billerica, MA, USA) using DMSO-*d*_6_ as solvents and TMS as an internal standard. Mass spectra were measured by Shimadzu GC–MS/QP (70 eV) (Shimadzu, Kyoto, Japan) CHN analyses were measured by Varia elemental analyzer III (Varia, Hanau, Germany). CHN analyses values were within ±0.4% of the theoretical values. All spectral analyses were done at the Micro-Analytical Center, Cairo, Egypt. Purity of the target derivatives was above 99.6%.

### 3.2. Synthesis of 6-Fluoro 2-(4-fluorophenyl)-benzo[d] [1,3] Oxazine-4-one *(**3**)*

This compound was prepared according to reported procedures [5]. 

Yield 80%; mp: 161–163 °C; IR (KBr, νmax, cm^−1^): 3052 (C–H), 1750 (C=O), 1518 (C=N), 1486 (C=C), 1369 (C–N). ^1^HNMR (DMSO-*d*_6_): δ 7.23–8.15 (m, 7H, Ar–H). ^13^CNMR (DMSO-*d*_6_): δ 110.1, 112.8, 116.9, 121.9, 125.9, 127.5, 130.9, 143.5, 156.7, 158.6, 164.2, 167.4. Anal. Calcd. For C_14_H_7_F_2_NO_2_ (259.04): C, 64.87; H, 2.72; N, 5.40. Found C, 64.66; H, 2.85; N, 5.61. MS (ESI) *m*/*z* 260.04 [M + 1].

### 3.3. Synthesis of 3-Amino-6-fluoro-2-(4-fluorophenyl)quinazolin-4(3H)-one *(**4**)*

This compound was prepared according to reported procedures [16]. 

Yield 40%; mp 230–232 °C; IR (KBr, νmax, cm^−1^): 3268 (s), 1572 (b) (NH_2_), 3079 (C–H), 1676 (C=O), 1568 (C=N), 1479 (C=C), 1372 (C–N). ^1^HNMR (DMSO-*d*_6_): δ 5.52 (s, 2H, NH_2_), 7.32–8.26 (m, 7H, Ar–H). ^13^C NMR (DMSO-*d*_6_): δ 112.7, 116.2, 119.9, 121.4, 123.7, 125.4, 127.8, 129.7, 146.4, 163.5, 165.9, 168.4. Anal. Calcd. For C_14_H_9_F_2_N_3_O (273.07): C, 61.54; H, 3.32; N, 15.38. Found C, 61.71; H, 3.51; N, 15.47. MS (ESI) *m*/*z* 274.07 [M + 1].

### 3.4. Synthesis of Substituted Quinazolinone Bearing Amino Acids *(**A**–**H**)*

3-amino 6-fluoro-2-(4-fluorophenyl)quinazolin-4(3*H*)-one (**4**), different Fmoc-protected l-amino acids and PyBOP in equimolar quantities (1 mmol) were dissolved in 30 mL anhydrous DMF. After fully dissolving, the reaction mixture was allowed to cool at 5–10 °C, then ten minutes later DIEA (1 mmol) was added. The reaction mixture was stirred at room temperature for 10 h. The solvent was removed by evaporation under high vacuum, then 20 mL of ethyl acetate was added to the residue, and the whole mixture was washed with 3 × 20 mL 1 N HCl 3 × 20 mL saturated sodium bicarbonate, and 3 × 20 mL brine. The organic layer was dried with sodium sulfate, filtered and concentrated to give the crude product. The crude product was dissolved in a 20 mL 20% piperidine solution in DMF, and allowed to stir overnight. DMF and piperidine were removed by evaporation under high vacuum. The remaining piperidine was removed by dilution with hexane and evaporation for five cycles until complete removal. The solid product was purified by recrystallization from ethanol to give the desired purified product.

*2-amino-N-(6-fluoro-2-(4-fluorophenyl)-4-oxoquinazolin-3(4H)-yl)acetamide* (**A**): Yield 55%; mp 236–238 °C; IR (KBr, νmax, cm^−1^): 3050 (CH), 1647 (C=O), 1538 (C=N), 1494 (C=C), 1378 (C–N). ^1^HNMR (DMSO-d_6_): δ 3.21 (s, 2H, CH_2_), 5.21 (s, 2H, NH_2_), 6.84–8.12 (m, 7H, Ar–H), 8.25 (s, 1H, NHCO). ^13^CNMR (DMSO-d_6_): δ 42.1, 112.6, 115.2, 121.7, 124.8, 128.9, 129.7, 146.5, 162.2, 163.1, 166.1, 166.9, 170.8. Anal. Calcd. For C_16_H_12_F_2_N_4_O_2_ (330.09): C, 58.18; H, 3.66; N, 16.96. Found C, 58.21; H, 3.78; N, 16.88. MS (ESI) *m*/*z* 331.09 [M + 1].

*(S)-2-amino-N-(6-fluoro-2-(4-fluorophenyl)-4-oxoquinazolin-3(4H)-yl)propanamide* (**B**): Yield 50%; mp 238–240 °C; IR (KBr, νmax, cm^−1^): 3055 (CH), 1665 (C=O), 1546 (C=N), 1485 (C=C), 1380 (C–N). ^1^HNMR (DMSO-d_6_): δ 1.32 (d, 3H, J = 5.3 Hz, CH_3_), 3.49 (q, H, J = 7.4, 7.8 Hz, CH), 5.27 (s, 2H, NH_2_), 6.98–8.21 (m, 7H, Ar–H), 8.42 (s, 1H, NHCO). ^13^CNMR (DMSO-d_6_): δ 20.8, 46.5, 116.2, 118.1, 119.8, 121.2, 124.6, 125.8, 127.5, 145.9, 161.9, 165.8, 166.9, 172.4. Anal. Calcd. For C_17_H_14_F_2_N_4_O_2_ (344.11): C, 59.30; H, 4.10; N, 16.27. Found C, 59.23; H, 4.23; N, 16.12. MS (ESI) *m*/*z* 345.11 [M + 1].

*(S)-2-amino-N-(6-fluoro-2-(4-fluorophenyl)-4-oxoquinazolin-3(4H)-yl)-3-methylbutanamide* (**C**): Yield 57%; mp 242–244 °C; IR (KBr, νmax, cm^−1^): 3051 (CH), 1662 (C=O), 1556 (C=N), 1475 (C=C), 1382 (C–N). ^1^HNMR (DMSO-d_6_): δ 1.12 (d, 6H, J = 5.4 Hz, 2CH_3_), 2.19 (d, H, J = 6.7 Hz, CH), 3.51 (d, H, J = 7.5 Hz, CH), 5.15 (s, 2H, NH_2_), 6.79–8.12 (m, 7H, Ar–H), 8.51 (s, 1H, NHCO). ^13^C NMR (DMSO-d_6_): δ 16.9, 31.5, 56.9, 114.6, 116.9, 120.3, 122.8, 126.2, 128.5, 129.6, 145.6, 162.6, 164.8, 168.5, 172.7. Anal. Calcd. For C_19_H_18_F_2_N_4_O_2_ (372.14): C, 61.28; H, 4.87; N, 15.05. Found C, 61.32; H, 4.95; N, 15.24. MS (ESI) *m*/*z* 373.14 [M + 1].

*(2S)-2-amino-N-(6-fluoro-2-(4-fluorophenyl)-4-oxoquinazolin-3(4H)-yl)-3-methylpentanamide* (**D**): Yield 55%; mp 248–250 °C; IR (KBr, νmax, cm^−1^): 3053 (CH), 1668 (C=O), 1557 (C=N), 1478 (C=C), 1381 (C–N). ^1^HNMR (DMSO-d_6_): δ 0.98 (t, 3H, J = 8.6 Hz, CH_3_), 1.06 (d, 3H, J = 5.6 Hz, CH_3_), 1.39–1.53 (m, 2H, CH_2_), 2.4–2.54 (m, H, CH), 3.51 (t, H, J = 7.8 Hz, CH), 5.31 (s, 2H, NH_2_), 6.96–8.15 (m, 7H, Ar–H), 8.33 (s, 1H, NHCO). ^13^CNMR (DMSO-d_6_): δ 11.2, 15.9, 26.5, 38.3, 56.7, 114.8, 118.1, 120.9, 122.6, 124.7, 127.5, 129.3, 145.3, 153.6, 162.5, 165.8, 169.5, 172.7. Anal. Calcd. For C_20_H_20_F_2_N_4_O_2_ (386.16): C, 62.17; H, 5.22; N, 14.50. Found C, 62.08; H, 5.17; N, 14.42. MS (ESI) *m*/*z* 387.16 [M + 1].

*(S)-4-(6-fluoro-2-(4-fluorophenyl)-4-oxoquinazolin-3(4H)-ylcarbamoyl)-4-aminobutanoic acid* (**E**): Yield 52%; mp 244–246 °C; IR (KBr, νmax, cm^−1^): 3057 (CH), 1671 (C=O), 1559 (C=N), 1478 (C=C), 1387 (C–N). ^1^HNMR (DMSO-d_6_): δ 2.13–2.32 (m, 4H, 2CH_2_), 3.47 (t, H, J = 7.8 Hz, CH), 5.4 (s, 2H, NH_2_), 6.87–8.06 (m, 7H, Ar–H), 8.37 (s, 1H, NHCO), 10.93 (s, 1H, COOH). ^13^CNMR (DMSO-d_6_): δ 26.2, 35.8, 54.7, 115.8, 118.1, 122.9, 123.5, 124.6, 126.7, 129.5, 144.8, 162.5, 164.1, 166.9, 169.2, 172.2, 176.9. Anal. Calcd. For C_19_H_16_F_2_N_4_O_4_ (402.11): C, 56.72; H, 4.01; N, 14.01. Found C, 56.68; H, 4.26; N, 14.12. MS (ESI) *m*/*z* 403.11 [M + 1].

*(S)-2-amino-N-(6-fluoro-2-(4-fluorophenyl)-4-oxoquinazolin-3(4H)-yl)-3-mercaptopropanamide* (**F**): Yield 52%; mp 220–222 °C; IR (KBr, νmax, cm^−1^): 3052 (CH), 1674 (C=O), 1558 (C=N), 1477 (C=C), 1383 (C–N). ^1^HNMR (DMSO-d_6_): δ 1.52 (t, H, J = 7.9 Hz, SH), 2.89 (t, 2H, J = 8.2 Hz, CH_2_), 3.57–3.71 (m, H, CH), 5.21 (s, 2H, NH_2_), 6.97–8.01 (m, 7H, Ar–H), 8.41 (s, 1H, NHCO). ^13^CNMR (DMSO-d_6_): δ 28.2, 56.9, 116.7, 118.9, 121.9, 124.5, 126.8, 128.7, 147.8, 162.6, 165.9, 172.2. Anal. Calcd. For C_17_H_14_F_2_N_4_O_2_S (376.08): C, 54.25; H, 3.75; N, 14.89. Found C, 54.41; H, 3.81; N, 14.72. MS (ESI) *m*/*z* 377.08 [M + 1].

*(S)-2-amino-N-(6-fluoro-2-(4-fluorophenyl)-4-oxoquinazolin-3(4H)-yl)-3-phenylpropanamide* (**G**): Yield 55%; mp 245–247 °C; IR (KBr, νmax, cm^−1^): 3059 (CH), 1677 (C=O), 1551 (C=N), 1476 (C=C), 1385 (C–N). ^1^HNMR (DMSO-d_6_): δ 2.98 (t, 2H, J = 8.1 Hz, CH_2_), 3.87–3.98 (m, H, CH), 5.36 (s, 2H, NH_2_), 6.64–8.21 (m, 12H, Ar–H), 8.51 (s, 1H, NHCO). ^13^CNMR (DMSO-d_6_): δ 42.1, 55.8, 116.6, 118.6, 120.5, 121.75, 124.1, 126.5, 127.4, 128.1, 129.7, 136.5, 139.6, 148.1, 161.2, 162.5, 166.9, 172. Anal. Calcd. For C_23_H_18_F_2_N_4_O_2_ (420.14): C, 65.71; H, 4.32; N, 13.33. Found C, 65.68; H, 4.45; N, 13.41. MS (ESI) *m*/*z* 421.14 [M + 1].

*N-(6-fluoro-2-(4-fluorophenyl)-4-oxoquinazolin-3(4H)-yl)pyrrolidine-2-carboxamide* (**H**): Yield 50%; mp 235–237 °C; IR (KBr, νmax, cm^−1^): 3050 (CH), 16711 (C=O), 1555 (C=N), 1472 (C=C), 1387 (C–N). ^1^HNMR (DMSO-d_6_): δ 1.67–2.86 (m, 7H, 3CH_2_, NH), 3.42 (t, H, J = 7.2 Hz, CH), 6.1–7.95 (m, 7H, Ar–H), 8.42 (s, 1H, NHCO). ^13^CNMR (DMSO-d_6_): δ 27.1, 33.9, 47.1, 64.7, 115.4, 117.8, 121.5, 122.8, 125.1, 126.8, 149.4, 158.5, 160.2, 162.2, 164.5, 165.9, 172.5. Anal. Calcd. For C_19_H_16_F_2_N_4_O_2_ (370.12): C, 61.62; H, 4.35; N, 15.13. Found C, 61.56; H, 4.41; N, 15.32. MS (ESI) *m*/*z* 371.12 [M + 1].

### 3.5. In Vitro Cytotoxic Screening 

Cells were cultured using reported culture media, incubated for 24 h, treated with different concentrations of tested derivatives and incubated for 48 h. MTT was added to extract the color, which was then measured by ELISA [12].

### 3.6. EGFR Inhibition Assay 

High tech HTScan EGFR kinase assay kits (Cell Signaling Technology, Danvers, MA, USA) were used to measure EGFR kinase activity. The assay was achieved following the manufacturers’ instructions and reported procedures [16,23]. 

### 3.7. Tubulin Polymerization Inhibition Assay

The influence of newly synthesized compound **E** on tubulin polymerization was detected kinetically using the CytoDYNAMIX Screen kit (BK006P, Cytoskeleton Inc., Denver, CO, USA). Cold porcine tubulin protein (>99% purity) was added to a G-PEM buffer (80 mM PIPES, 2 mM MgCl_2_, 0.5 mM EGTA, 1 mM GTP, pH 6.9) containing 15% glycerol with or without the identified compounds. The sample mixture was dotted onto a pre-warmed 96-well plate, which was directly moved to a 37 °C plate reader (SpectraMax Plus, Molecular Devices Inc., Sunnyvale, CA, USA). The absorbance was monitored every minute for 30 min at 340 nm [16,23].

### 3.8. Molecular Docking

Molecular Operating Environment (MOE) software was used to perform molecular docking [27]. Newly synthesized derivatives were docked into the 3D form of the two active targets: The crystal structures of EGFR (PDB code: 1M17) complexes with erlotinib at 2.6 Å resolution; and the crystal structure of tubulin (PDB: 1SA0) complexes with colchicine at 3.5 Å resolution [28]. The projected binding of the target derivatives to the active pocket of each EGFR and tubulin complex was determined as the best classified scoring function, representing the conformational structures with the most favorable binding energy (ΔE). The data of binding energies and scores was used to calculate the binding affinity of all docked derivatives.

### 3.9. Statistical Analysis

Experiments were achieved in triplicate. The results were shown as the mean ± SD (standard deviation) using the student’s T method.

## 4. Conclusions

New fluoroquinazolinones (**A**–**H**) were designed, prepared and tested as antitumor agents against MCF-7 and MDA-MBA-231 cancer cells. These compounds presented good antitumor activity, ranging from 0.43 ± 0.02 µM to 68.49 ± 3.27 µM against the two cell lines. Two derivatives, **E** and **G**, showed higher antitumor activity than erlotinib on the two cell lines. An EGFR assay of these two highly active derivatives exhibited excellent activity in comparison to erlotinib. Compound **E** was tested as a tubulin inhibitor and compared with colchicine and displayed a good result. Molecular docking of the highest active compound, compound **E**, correlated with the experimental results and elucidated the mode of binding of these derivatives with the EGFR and tubulin binding sites. The mode of binding showed favorable ligand–receptor interactions and extra hydrogen bonds with the receptor sites. The best active derivatives, **E** and **G**, may be exposed to future modifications and exploration in order to become active antitumor drugs.

## Figures and Tables

**Table 1:** IC_50_ values for antitumor screening of target derivatives. The data shown are the mean ± standard deviation (SD) of at least three independent experimentations.

Compound	IC_50_ (µM)		3rd Position Substitution
	MCF-7	MDA-MBA-231	
**A**	24.97 ± 1.61	16.34 ± 1.21	Glycine
**B**	68.49 ± 3.27	42.93 ± 2.64	l-alanine
**C**	1.16 ± 0.05	3.45 ± 0.21	l-valine
**D**	11.28 ± 1.25	7.49 ± 0.42	l-isoleucine
**E**	12.44 ± 5.73	0.43 ± 0.02	l-glutamine
**F**	1.93 ± 0.08	12.46 ± 5.88	l-cysteine
**G**	0.44 ± 0.01	24.67 ± 1.7	l-phenylalanine
**H**	1.28 ± 0.03	11.96 ± 1.33	l-proline
**Erlotinib**	1.14 ± 004	2.55 ± 0.19	

**Table 2:** Selectivity of the target derivatives organized according to type of selectivity. S1 = IC_50_ (MCF-7)/IC_50_ (MDA-MBA-231), however S2 = IC_50_ (MDA-MBA-231)/IC_50_ (MCF-7). When S1 > S2, the compound is more selective to MDA-MBA-231, and when S2 > S1, the compound is more selective to MCF-7. Data shown are the mean values ± SD from at least three experiments.

Compound	Selectivity Indices		Cell Line
	S1	S2	
**E**	28.93 ± 0.08	0.03 ± 0.11	MDA-MBA-231 selective
**B**	1.59 ± 0.05	0.63 ± 0.23	
**A**	1.53 ± 0.12	0.65 ± 0.05	
**D**	1.51 ± 0.24	0.66 ± 0.17	
**Erlotinib**	0.45 ± 0.09	2.24 ± 0.31	MCF-7 selective
**C**	0.34 ± 0.18	2.98 ± 0.71	
**F**	0.15 ± 0.17	6.46 ± 0.04	
**H**	0.11 ± 0.23	9.34 ± 0.02	
**G**	0.02 ± 0.07	56.07 ± 0.15	

**Table 3:** IC_50_ values for the epidermal growth factor receptor (EGFR) assay of compounds **G**, **E** and erlotinib. Data shown are the mean values ± SD from at least three experiments.

Compound	IC_50_ (nM)		Amino Acid at Position 3
	MCF-7	MDA-MBA-231	
**G**	163.97 ± 0.07	-	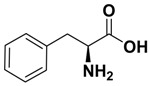
**E**	-	545.38 ± 0.04	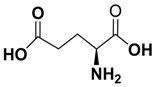
**Erlotinib**	78.04 ± 0.12	299 ± 0.35	

**Table 4:** Docking scores and energy values of target compounds and reference erlotinib complexes with EGFR (PDB; 1M17) using the Molecular Operating Environment (MOE) software.

Compound	Score	ΔE (Kcal/mol)
**A**	−5.4	−9.8
**B**	−4.1	−8.7
**C**	−6.8	−12.2
**D**	−7.2	−11.8
**E**	−15.3	−23.5
**F**	−6.42	−10.7
**G**	−11.6	−17.4
**H**	−6.9	−10.1
**Erlotinib**	−8.7	−13.6

**Table 5:** Docking scores and energy values of target compounds and reference colchicine complexes with tubulin (PDB; 1SA0), using the MOE software.

Compound	Score	ΔE (Kcal/mol)
**A**	–9.6	–12.4
**B**	–6.8	–11.5
**C**	–10.4	–12.5
**D**	–8.1	–13.2
**E**	–15.4	–24.7
**F**	–7.2	–11.2
**G**	–9.1	–15.3
**H**	–7.2	–10.5
**Colchicine**	–9.3	–11.1
